# Dihydromyricetin functions as a tumor suppressor in hepatoblastoma by regulating SOD1/ROS pathway

**DOI:** 10.3389/fonc.2023.1160548

**Published:** 2023-05-15

**Authors:** Tong Guo, Xitong Wang, Gensheng Zhang, Tian Xia, Runzhi Zhu, Jinfa Tou

**Affiliations:** ^1^ Department of Neonatal Surgery, The Children’s Hospital, Zhejiang University School of Medicine, National Clinical Research Center for Child Health, Hangzhou, China; ^2^ National Clinical Research Center for Child Health, The Children’s Hospital, Zhejiang University School of Medicine, Hangzhou, Zhejiang, China

**Keywords:** apoptosis, hepatoblastoma, SOD1, ROS, anticancer activity

## Abstract

**Background:**

Hepatoblastoma has an unsatisfactory prognosis, and traditional chemotherapy has strong side effects. Dihydromyricetin is a flavonoid extracted from a woody vine of the genus Serpentine in the family Vitaceae, with effects such as preventing alcoholic liver and reducing the incidence of liver cancer. However, the effect of DHM on hepatoblastoma and its specific pathway are still unclear.

**Purpose:**

The purpose of this study was to investigate the effects of DHM on children's hepatoblastoma and its related mechanisms.

**Methods:**

CCK-8 assays were used to measure proliferation. Apoptosis and reactive oxygen species (ROS) were analyzed by flow cytometry. Apoptotic cells were observed using Hoechst 33342 staining and fluorescence microscopy. Protein expression levels in HuH-6 and HepG2 cells were determined by western blotting.

**Results:**

We found that DHM was able to inhibit the growth and increase cellular mortality in HuH-6 and HepG2 cells. Furthermore, DHM decreased the intracellular ROS level and increased the expression of SOD1. ROS scavenger NAC promoted apoptosis, while the use of SOD1 inhibitor LCS-1 weakened the ROS scavenging effect of DHM , and to some extent reduced the killing effect of DHM on hepatoblastoma cells.

**Conclusion:**

These results suggest that regulating SOD1/ROS pathway to induce apoptosis is one of the potential mechanisms of DHM as a tumor suppressor in hepatoblastoma. Therefore, DHM may be a novel candidate for inhibiting hepatoblastoma growth and deserves further study.

## Introduction

Hepatoblastoma is most common in infants under three years old, and its incidence has been increasing in the past thirty years (1). Surgery is currently that basic treatment for hepatoblastoma (2). But surgical treatment of hepatoblastoma alone cannot achieve satisfactory therapeutic results. Neoadjuvant chemotherapy is beneficial to the surgical resection of hepatoblastoma, which greatly increases the chances of complete resection of the tumor (3). However, chemotherapy drugs generally cause adverse reactions, such as hearing loss and cardiotoxicity, which bring great harm to children’s physical and mental health (4, 5). Therefore, it is imperative to develop novel medicine with fewer side effects for treating children’s hepatoblastoma.

DHM is a plant extract with anti-oxidation, liver protection, anti-inflammatory and anti-tumor properties (6–9). Previous reports have proved that DHM has anticancer effects in a variety of tumors, including liver carcinoma, gastric cancer and lung cancer (10–12). However, whether DHM has a therapeutic effect on hepatoblastoma is unknown.

Apoptosis is a programmed form of cells death, which can clear the damaged and redundant cells in time to maintain the steady state of tissues, organs and internal environment (13). Apoptosis is often accompanied by typical changes, such as cell volume reduction, cell permeability change and chromatin condensation (14). The ability of tumor cells to escape apoptosis is regarded as a hallmark of cancer (15). Therefore, inhibiting the evasion of apoptosis may be a feasible treatment to inhibit hepatoblastoma growth.

ROS are natural byproducts produced by aerobic cells in metabolic processes and play a significant role in tumor cells apoptosis (16). Cellular growth and survival require a certain level of ROS (17). ROS levels below a certain threshold will trigger apoptosis (18). Under relatively low conditions, ROS act as cytokines and growth factors and are essential second messengers to promote cell proliferation and survival (19, 20). High ROS levels may cause mitochondrial dysfunction, thus inducing the release of Cyt C and, ultimately, leading to apoptosis (21). Thus, most antitumor agents exert antitumor effects by increasing ROS levels above the toxic threshold (22). SOD1 is a vital antioxidant enzyme in the cytoplasm of organisms, which can efficiently remove the intracellular ROS level (23).

## Materials and methods

### Chemicals and reagents

DHM was purchased from Meica (China), dissolved in dimethyl sulfoxide (DMSO) and stored at -20°C for less than two months. The stock solutions were diluted to the required concentrations in DMEM (Gibco) medium. The final DMSO concentration was always less than 0.2% (v/v). The antibodies used in this study included Bcl-2 (15071S, CST), Bax (5023S, CST), cleaved PARP (5625S, CST), cleaved caspase-3 (9661S, CST), cleaved caspase-9 (20750S, CST), Cytochrome c (4280S, CST) and β-Actin (4970S, CST). All primary antibodies were diluted at a ratio of 1:1,000. Goat anti-mouse IgG (H + L) HRP (FDM007), goat anti-rabbit IgG (H + L) HRP (FDR007) and the FDbio-Dura ECL Kit (FD8020) were purchased from Fudebio (Hangzhou, China). The secondary antibodies were diluted 1:5000. PageRuler Prestained Protein Ladder was purchased from Thermo Fisher Scientific. The CCK-8 kit (CA1210-500) was purchased from Solarbio (Beijing, China). The ROS assay kit (S0033M), Hoechst 33342, and NAC were obtained from Beyotime Biotechnology. The working concentration of NAC is 10 µM, and the pretreatment time is 2h. Apoptosis assay kits were obtained from BD Company (item 556547). LCS-1 was purchased from MedChem Express (MCE, USA) and used at a final working concentration of 1 µM. Cisplatin was purchased from Selleck (Cat. No. S1166).

### Cell lines and culture

HuH-6 and HepG2 cells were obtained from ATCC. The cells were grown in DMEM Medium supplemented with 10% FBS (Capricorn) and 1% penicillin/streptomycin (Sigma) at 37°C with 5% CO_2_.

### Cell viability assay

Cell counting kit -8 (CCK-8) is a widely used cell viability assay that measures the metabolic activity of cells by detecting the reduction of a tetrazolium salt (WST-8) to a highly colored formazan dye (24). CCK-8 has higher sensitivity and a better reproducibility than traditional MTT assay (25). In this study, CCK-8 assay was used to evaluate the viability of HuH-6 and HepG2 cells treated with DHM or other reagents. The concentration gradient of DHM was 0, 75, 150 µM (HuH-6 cells) or 0, 50, 100 µM (HepG2 cells), and the treatment time was 24h, 48h and 72h. The working concentration of LCS-1 is 1 µM, and the treatment time is 24h. The working concentration of NAC is 10 µM, and the pretreatment time is 2h. The cells were cultured in different concentrations of DHM or other experimental reagents for specific time. Before analysis, add 10 µL CCK-8 reagent into each well and incubate at 37°C for 2h. Finally, the optical density (OD) was detected by microplate reader.

### Hoechst 33342 staining

Apoptosis was visualized by microscopic examination of cells that were stained with Hoechst 33342. The cells were cultured with DHM for 24 h. The concentration gradient of DHM was 0, 75, 150 µM (HuH-6 cells) or 0, 50, 100 µM (HepG2 cells). Subsequently, the cells were stained with Hoechst 33342 at 37°C for about 20 minutes. Then the stained cells were then visualized by fluorescence microscopy.

### Flow cytometric analysis of apoptosis

The cells were cultured in different concentrations of DHM or other experimental reagents for specific time. The concentration gradient of DHM was 0, 75, 150 µM (HuH-6 cells) or 0, 50, 100 µM (HepG2 cells), and the treatment time was 24h. The working concentration of LCS-1 is 1 µM, and the treatment time is 24h. The working concentration of NAC is 10 µM, and the pretreatment time is 2h. The cells were digested using pancreatic enzymes without EDTA. After the dark-light staining with FITC-AnnexinV and PI for 15 min, the apoptotic cells were analyzed by flow cytometry (BD FACSLyric).

### Western blotting

The concentration gradient of DHM was 0, 75, 150 µM (HuH-6 cells) or 0, 50, 100 µM (HepG2 cells), and the treatment time was 24h. HuH-6 and HepG2 cells were treated with DHM, and then lysed with RIPA buffer at 4°C for 30 minutes to extract the target protein. The protein samples of each group were separated by 4-12% ExpressPlus PAGE Gels (Genscript Biotech Corporation, China), and then transferred to PVDF membrane. Then the PVDF membrane was incubated with primary and secondary antibodies. Finally, the PVDF membranes were developed with the FDbio-Dura ECL kit (FD8020).

### Measurement of intracellular ROS generation

An ROS assay kit was used to measure the intracellular ROS in HuH-6 and HepG2 cells. The concentration of DHM was 75 µM (HuH-6 cells) and 50 µM (HepG2 cells), and the treatment time was 12h. The working concentration of LCS-1 is 1 µM, and the treatment time is 12h. The working concentration of NAC is 10 µM, and the pretreatment time is 2h. Briefly, the cells were treated with DHM, NAC or LCS-1 for 12h and then incubated with working concentrations of DCHF-DA (Beyotime Biotechnology) at 37°C for 20 minutes. Finally, the fluorescence intensity representing the amount of intracellular ROS was detected by flow cytometry (BD FACSLyric).

### Statistical analysis

The experiments were repeated at least three times, and the data are expressed as the mean ± standard deviation (SD) of the three repeated experiments. All statistical analyses were performed using GraphPad Prism 9. T test was used to compare the differences between the two groups. The analysis of differences among multiple groups was performed by one-way ANOVA, followed by Tukey’s or Dunnett’s multiple comparisons test. Unless stated otherwise, statistical significance is displayed as ∗P < 0.05, ∗∗P < 0.01, ∗∗∗P < 0.001, and ∗∗∗∗P < 0.0001.

## Results

### DHM inhibits cell viability in hepatoblastoma cells

**Figure 1:**
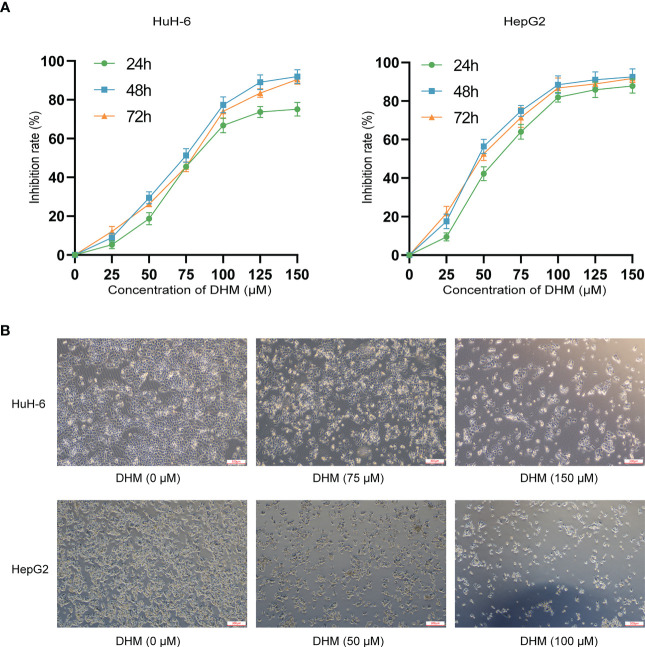
DHM inhibits the proliferation and growth of human hepatoblastoma HuH-6 and HepG2 cells. **(A)** HuH-6 and HepG2 cell proliferation was measured by CCK-8 assays. **(B)** The morphological characteristics of HuH-6 and HepG2 cells after treatment with different concentrations of DHM.

CCK-8 (Cell Counting Kit-8) assay is a cell viability assay used for measuring cell proliferation or cytotoxicity. We used CCK-8 experiment to evaluate the cell viability to demonstrate the impairment of DHM on the growth of hepatoblastoma cells. As shown in **Figure 1A**, compared with untreated cells, DHM significantly reduced the vitality of hepatoblastoma cells, and these effects were positively correlated with the concentration of DHM. When treated with DHM for 24 hours, the semi-inhibitory concentrations of HuH-6 and HepG2 cells were about 75.67 μM and 43.32 μM, respectively. After 48 h treatment with 75 μM DHM, the inhibitory rate of HuH-6 cells was 48.08 ± 2.83%. Similarly, when treated with 50 μM DHM for 48 h, the inhibition rate of HepG2 cells was 54.77 ± 5.02%. Subsequently, we examined the impact of DHM on HuH-6 and HepG2 cells morphology by optical microscopy. After DHM treatment, the cells became round and shrunken, and cell numbers decreased compared to untreated group, as shown in **Figure 1B**. These results are consistent with those obtained from the CCK-8 assays, which suggests that DHM inhibited the growth and division of hepatoblastoma cells.

### DHM induces apoptosis in hepatoblastoma cells

**Figure 2.1:**
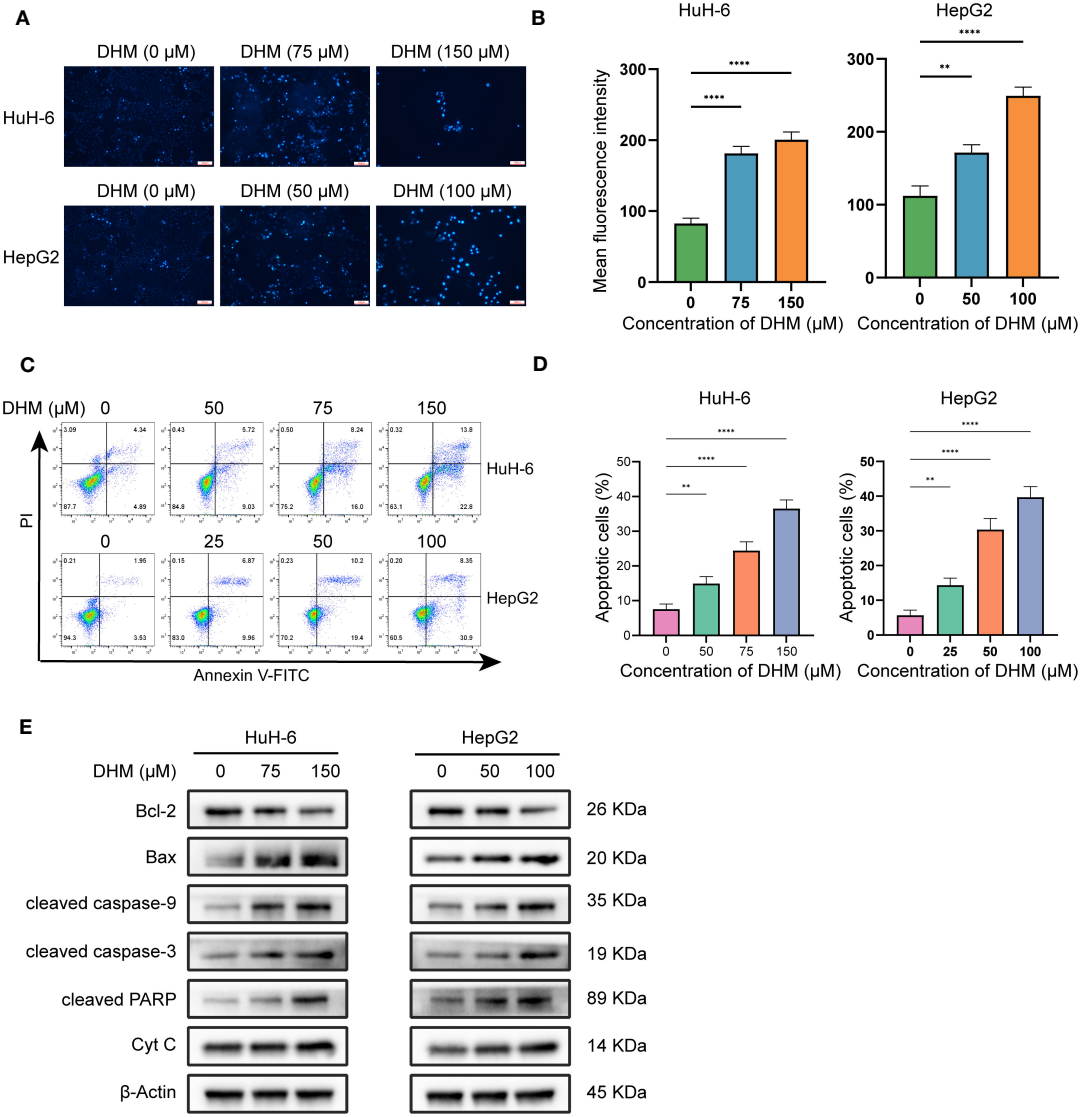
DHM induces apoptosis in HuH-6 and HepG2 cells. **(A)** Apoptotic cell death was evaluated by Hoechst 33342 staining (Scale bars = 300 μM). **(B)** The fluorescence intensity of cells stained with Hoechst33342 was analyzed by ImageJ. The data are expressed as the mean ± standard deviation (SD) of the three repeated experiments. **(C)** HuH-6 and HepG2 cells were exposed to DHM for 24 h. Annexin V-FITC/PI staining was used for the apoptosis assay. **(D)** The histograms show the percentage of apoptotic cells, and the data represent the mean ± SD of three independent experiments. **(E)** The expression levels of Bax, Bcl-2, cleaved caspase-3, cleaved caspase-9, cleaved PARP and Cyt C were measured by western blotting. β-Actin is shown as the loading control. HuH-6 and HepG2 cells were treated with DHM for 24 h. Statistical significance is displayed as ∗∗P < 0.01, and ∗∗∗∗P < 0.0001.

In this study, we confirmed that DHM could induce the apoptosis of hepatoblastoma cells by analyzing the changes of Hoechst 33342 staining, apoptosis rate and expression level of apoptosis-related proteins after DHM treatment. Firstly, we confirmed the apoptosis by Hoechst 33342 staining. Normal cell membrane only allows a small amount of Hoechst 33342 to enter, so it appears light blue under microscope. When cells undergo apoptosis, the permeability of cell membrane will be enhanced, resulting in more dyes combining with DNA and producing bright blue fluorescence. Therefore, the level of blue fluorescence under the microscope can be used to judge whether the cells have apoptosis. As shown in **Figures 2.1A, B**, most of the cells in the control group showed weak blue fluorescence, while the cells treated with DHM showed strong blue fluorescence, indicating that these cells had apoptosis under the influence of DHM. We next examined the changes in the apoptosis rate after DHM treatment. In **Figure 2.1C**, the up-right quadrant represents late apoptotic cells or dead cells, and the low-right quadrant represents early apoptotic cells. We count the sum of early apoptosis and late apoptosis as the apoptosis rate, which is used to indicate the killing effect of drugs on cells. Our experimental results show that the apoptosis rate of HuH-6 treated with 75 μM DHM was 24.41 ± 2.50%, while that of control cells was 7.58 ± 1.54%. The apoptosis rate of HepG2 treated with 50 μM DHM was 30.33 ± 3.21%, and that of the corresponding control group was 5.67 ± 1.53% (**Figures 2.1C, D**).

To further explore the specific mechanism of DHM inducing HuH-6 and HepG2 cell death, we detected the amount of intracellular Bcl-2 family proteins by western blot, which are important regulatory factors in cell apoptosis. The results showed that as DHM concentration increased, Bax levels increased while Bcl-2 levels decreased. Bax and Bcl-2 can regulate the release of cyt c, and then control the cascade activation of caspase. Caspase cascade activation is one of the most critical processes in apoptosis mediated by mitochondrial pathway (26). Therefore, we next studied the changes of the amount of molecules related to apoptotic signals. As shown in **Figure 2.1E**, with the increase of DHM concentration, the activation of caspase-9, caspase-3 and PARP was enhanced. In addition, the expression of cyt c in the cytoplasm of HuH-6 and HepG2 cells was also up-regulated. These results suggest that DHM can regulate the expression levels of proteins associated with apoptosis, which in turn leads to apoptosis of HuH-6 and HepG2 cells.

**Figure 2.2:**
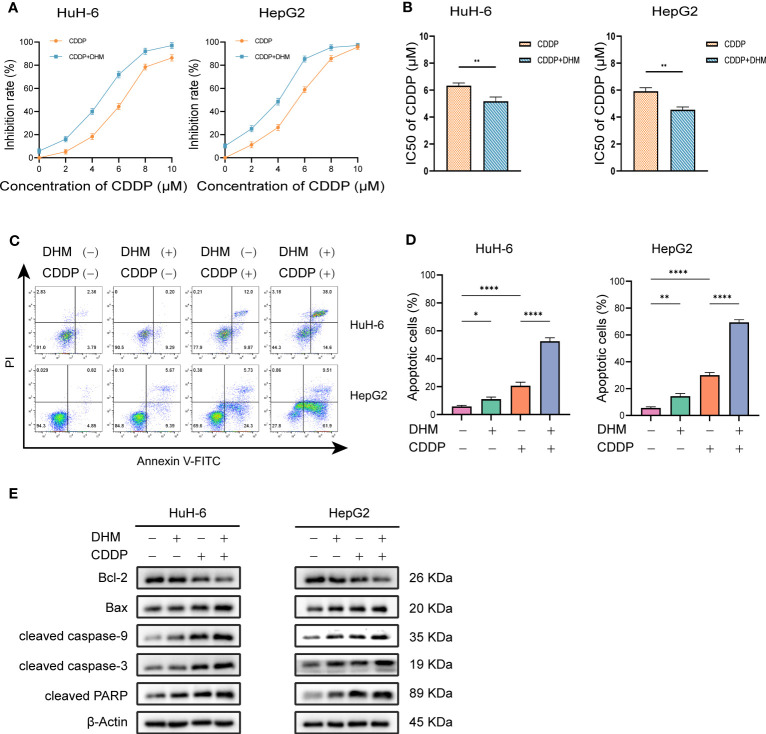
**(A)** HuH-6 and HepG2 cells were treated with cisplatin (0, 2, 4, 6, 8 µM) alone or in combination with DHM (25 µM) for 24 hours, and the cell viability was detected by CCK-8 assay. **(B)** The IC50 was calculated by GraphPad Prism 9.0 software, and the differences between the two groups were compared by t-test. **(C)** HuH-6 and HepG2 cells were treated with cisplatin (6 µM) alone or in combination with DHM (25 µM) for 24 hours, and the apoptosis rate of HuH-6 and HepG2 cells was detected by flow cytometry. **(D)** The percentage of apoptotic cells was statistically analyzed by GraphPad Prism 9 software. Data are represented as mean ± SD obtained from three independent experiments. **(E)** HuH-6 and HepG2 cells were treated with cisplatin (6 µM) alone or in combination with DHM (25 µM) for 24 hours. The expression levels of Bax, Bcl-2, cleaved caspase-3, cleaved caspase-9 and cleaved PARP were measured by western blotting. Statistical significance is displayed as ∗P < 0.05, ∗∗P < 0.01, and ∗∗∗∗P < 0.0001.

Cisplatin (cis-dichlorodiammine platinum, CDDP) is a common chemotherapy drug for hepatoblastoma. We studied the inhibitory effect of DHM combined with cisplatin on HB cells. We used a low concentration of DHM (25 µM) for this experiment. As shown in **Figure 2.2A**, compared with cisplatin alone, the combination of DHM (25 µM) and cisplatin can significantly improve the inhibition rate of HuH-6 and HepG2 cells. In HuH-6 cells, the semi-inhibitory concentration (IC50) of cisplatin alone was 6.33 ± 0.20 µM, and that of cisplatin combined with DHM was 5.17 ± 0.31 µM. In HepG2 cells, the IC50 of cisplatin alone was 5.91 ± 0.27 µM, and that of cisplatin combined with DHM was 4.54 ± 0.21 µM (**Figure 2.2B**). Therefore, DHM can reduce the IC50 of cisplatin in hepatoblastoma. In addition, the combination with DHM can significantly improve the killing effect of cisplatin on HuH-6 and HepG2 cells (**Figures 2.2C, D**). Our subsequent WB experiments showed that compared with cisplatin alone, DHM significantly increased the expression of Bax and the activation of caspase-9, caspase-3, and PARP, and significantly reduced the expression of Bcl-2 (**Figure 2.2E**). These results indicate that DHM could enhance the inhibitory effect of cisplatin on HuH-6 and HepG2 cells.

### DHM downregulates ROS in hepatoblastoma cells by targeting SOD1

**Figure 3:**
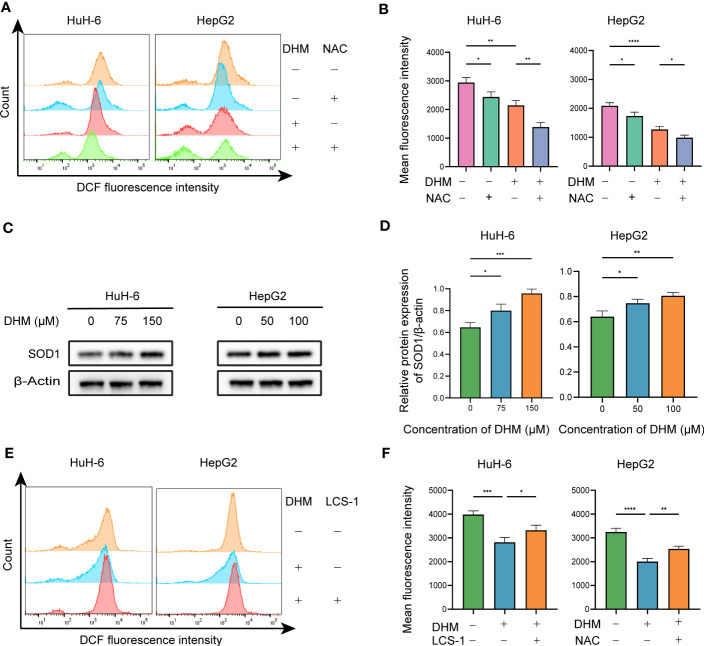
DHM downregulates ROS in hepatoblastoma cells by targeting SOD1. **(A)** Intracellular ROS levels were assessed by determining DCF fluorescence intensity *via* flow cytometry. The working concentration of NAC is 10 µM, and the pretreatment time is 2h. **(B)** Quantitative analysis of the mean fluorescence intensity of DCF. Data are represented as mean ± standard deviation (SD) obtained from three independent experiments. **(C)** The expression levels of SOD1 were measured by western blotting. HuH-6 and HepG2 cells were treated with DHM for 24 h **(D)** Bands were analyzed with ImageJ software, normalized to β-actin, and expressed relative to the control group. **(E)** Intracellular ROS levels were assessed by determining DCF fluorescence intensity *via* flow cytometry. The working concentration of LCS-1 is 1 µM. **(F)** Quantitative analysis of the mean fluorescence intensity of DCF. The bars represent the means ± SDs of three independent experiments. statistical significance is displayed as ∗P < 0.05, ∗∗P < 0.01, ∗∗∗P < 0.001, and ∗∗∗∗P < 0.0001.

DHM is a flavonoid compound that can scavenge ROS. We further examined whether the inhibition of DHM on hepatoblastoma was related to its ability to scavenge ROS. After 12 hours of DHM administration, DHM significantly reduced ROS levels in HuH-6 and HepG2 cells compared with controls. In addition, to demonstrate that NAC acts as a ROS scavenger, we pretreated cells with NAC, which resulted in significant decreases in ROS levels in these cells (**Figures 3A, B**). SOD1 is one of the most important enzymes to reduce the level of ROS and plays a key role in maintaining the ROS homeostasis (27). To confirm whether DHM reduces intracellular ROS levels by up-regulating SOD1, we first tested the effect of DHM on the expression of SOD1 protein by western blot. As shown in the **Figures 3C, D**, DHM upregulated the level of SOD1 in both HuH-6 and HepG2 cells. Next, we examined the effect on ROS levels following inhibition of SOD1. As shown in the **Figures 3E, F**, the use of LCS-1 weakened the extent to which DHM reduced ROS in HuH-6 and HepG2 cells. The above results have demonstrated that DHM reduced intracellular ROS by up-regulating SOD1.

### The downregulation of ROS by DHM is responsible for cell proliferation and apoptosis

**Figure 4:**
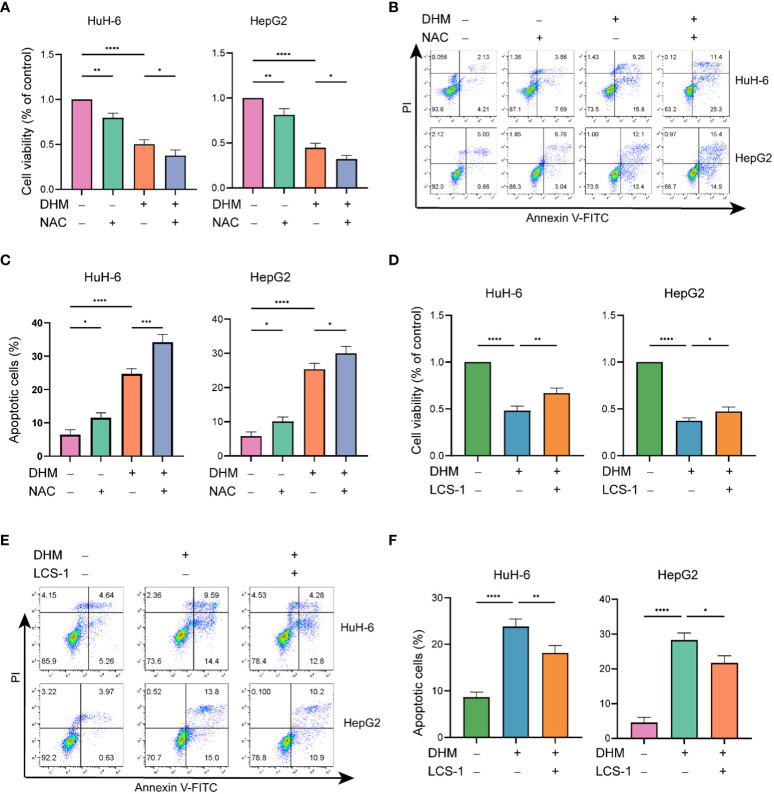
The downregulation of ROS by DHM is responsible for cell proliferation and apoptosis. **(A)** Viability of the DHM/NAC-treated cells were assayed by CCK-8 assay. The working concentration of NAC is 10 µM, and the pretreatment time is 2h. **(B)** Analysis of apoptotic HuH-6 and HepG2 cells by flow cytometry after treatment with DHM/NAC. The working concentration of NAC is 10 µM, and the pretreatment time is 2h. **(C)** The histograms show the percentage of apoptotic cells, and the data represent the mean ± SD of three independent experiments. **(D)** CCK-8 assay was performed to check the cell viability after respective experimental treatments. The working concentration of LCS-1 is 1 µM. **(E)** Following the corresponding treatment, apoptotic rates were measured by flow cytometry. The working concentration of LCS-1 is 1 µM. **(F)** The histograms show the percentage of apoptotic cells, and the data represent the mean ± SD of three independent experiments. statistical significance is displayed as ∗P < 0.05, ∗∗P < 0.01, ∗∗∗P < 0.001, and ∗∗∗∗P < 0.0001.

We further investigated whether the killing effect of DHM on hepatoblastoma cells is related to its ROS scavenging effect. As shown in **Figures 4A–C**, compared with the control group, the cell viability of the group pretreated with NAC was significantly decreased, and the apoptotic cells were increased. Therefore, scavenging intracellular ROS levels may be one of the ways for DHM to inhibit the growth of hepatoblastoma cells and promote the apoptosis of tumor cells. Next, we examined the changes in proliferation and apoptosis of hepatoblastoma cells after inhibition of SOD1 with LCS-1. As shown in **Figures 4D–F**, LCS-1 significantly attenuated the effect of DHM in inhibiting cell proliferation and inducing apoptosis. The above results suggest that SOD1 is involved in the role of DHM in reducing ROS level, which is one of the potential ways for DHM to inhibit the proliferation and induce apoptosis of hepatoblastoma cells.

## Discussion

Hepatoblastoma is a solid tumor in children with high malignancy and high mortality (28). Surgery and chemotherapy are the primary methods to treat hepatoblastoma, but many chemotherapy drugs used for hepatoblastoma have strong side effects at present (29). DHM is a natural active product extracted from a vine of Ampelopsis of Vitaceae, which has many biological activities such as anti-inflammatory, anti-oxidation and anti-tumor (30–33). Previous studies have shown that DHM plays an anti-tumor role in many tumors, including human lung cancer, liver cancer, gastric cancer, melanoma, osteosarcoma and ovarian cancer. Its potential mechanisms include inhibiting cell proliferation, inducing cell cycle arrest, inducing tumor cells to migrate and invade, and inhibiting angiogenesis (34–38). In addition, many studies show that DHM has a good therapeutic effect on alcoholic liver injury, fatty liver, liver fibrosis and drug-induced hepatotoxicity (39–43). Considering the protective effect and anti-tumor activity of DHM, we speculate that DHM may be used as an adjuvant therapy for hepatoblastoma in children.

Apoptosis is a process of self-extinction of cells, therefore, it is also one of the bases for antitumor drugs to treat tumors clinically. The antiapoptotic protein Bcl-2 is predominantly localized in mitochondria, preventing Cyt C release from mitochondria and leading to the inactivation of critical caspase (44). Bax, a protein that promotes apoptosis, is mainly present in the cytosol in nonapoptotic cells, and it translocates to mitochondria following an apoptotic stimulus, releasing Cyt C into cytoplasm. The transferred Cyt C can further bind to caspase-9 and APAF1 to form apoptotic bodies, thereby activating caspase-3 (45). Cleaved caspase-3 cleaves its corresponding substrate PARP, causing PARP to lose its ability to repair DNA and leading to apoptosis (46). Therefore, cleaved caspase-3 and cleaved PARP are recognized as the markers of apoptosis. In our study, with the increase of DHM concentration, Bcl-2 displayed a downward trend, while Bax showed an upward trend, which was a sign of increased apoptosis. In addition, the activation of caspase-9, caspase-3 and PARP also showed a positive correlation with DHM concentration. Thus, it can be concluded that DHM regulated the expression of apoptosis-related proteins and caused the apoptosis of HuH-6 and HepG2 cells.

Extensive evidence suggests that ROS play crucial roles in multiple links of tumor initiation and progression. Compared to normal healthy cells, tumor cells usually need higher levels of ROS to promote tumorigenesis and tumor development (47). If the level of intracellular ROS is below the basic requirements for cell growth, cancer cells cannot proliferate normally (48). It has been shown that the decreased ROS levels can induce apoptosis by releasing proapoptotic proteins and Cyt C (49). In this study, pretreatment with NAC decreased cell viability and increased apoptosis rates compared with those in the control group. Therefore, scavenging intracellular ROS may be one of the mechanisms by which DHM suppresses proliferation and promotes apoptosis in HuH-6 and HepG2 cells. Although further research is needed to prove the molecular mechanism of DHM reducing intracellular ROS level in hepatoblastoma, our data show that ROS clearance by DHM is one of the pathways that inhibit hepatoblastoma. There is another interesting implication of this result. Currently, many anticancer drugs treat tumors by increasing ROS to toxic levels, which leads to harmful side effects (50). We demonstrated that DHM reduced ROS levels in hepatoblastoma cells, thus avoiding killing normal cells when exerting its antitumor effects.

SOD1 is an important antioxidant enzyme of organisms, whose main role is to remove intracellular superoxide radicals (51). Studies have shown that SOD1 can be widely expressed in cells, down-regulating the peroxides in tumor cells to extremely low levels, thereby leading to imbalance of energy metabolism in tumor cells and eventually resulting in cell death (52). Additional studies have shown that upregulation of SOD1 can potentially be used to induce cancer cell death (53). However, the mechanism of anti-tumor by regulating SOD1 signaling pathway has not been fully clarified. Our research shows that the targeted inhibition of SOD1 can increase the intracellular ROS level of HuH-6 and HepG2 cells, which proves that SOD1 plays a potential role in eliminating intracellular ROS. At the same time, inhibition of SOD1 weakened the inhibitory effect of DHM on hepatoblastoma. It can be concluded that the regulation of SOD1/ROS signaling pathway by DHM is involved in DHM’s inhibition of proliferation and induction of apoptosis in hepatoblastoma cells.

## Conclusions

In this study, we confirmed that DHM inhibited the growth of hepatoblastoma cells and induced apoptosis through down-regulation of ROS by targeting SOD1. In addition, contrary to common chemotherapy drugs, the pharmacological mechanism of DHM is to reduce ROS production in tumor cells rather than increase it. Therefore, it can be demonstrated that the important advantage of DHM is that its use to treat hepatoblastoma will not cause serious side effects to normal cells of the body. In conclusion, our research proves that DHM has the potential to treat hepatoblastoma as a new natural antitumor medicine.

## Data availability statement

The raw data supporting the conclusions of this article will be made available by the authors, without undue reservation.

## Author contributions

TG was responsible for the main experiments and wrote the manuscript. XW was responsible for collection and analysis of data. GZ and TX participated in experimental design. RZ and JT are responsible for reviewing and adjusting this paper. All authors contributed to the article and approved the submitted version.
